# Buttermilk in Infant Feeding

**Published:** 1908-12-19

**Authors:** I. Graanboom

**Affiliations:** Amsterdam, Weteringschans 98


### Editor's Letter-Box.

**Our correspondents are reminded that prolixity is a great bar to publication, and that brevity of style and conciseness of statement greatly facilitate early insertion.**

### BUTTERMILK IN INFANT FEEDING.

Sir,—With reference to an article which appeared in The Hospital on August 15, 1908, I should like to point out the results obtained here in the treatment of more than 1,000 babies with buttermilk. These were partly hospital cases and partly in private practice, and they were reported upon fully in the *Revue d'Hygiene et de medicine infantile*, 1904, p. 433, and *Nederlandsche Tydschrift voor Geneeskunde*, 1906, p. 623. The experience gained coincides with the excellent results obtained by other Dutch authorities, particularly Teixeira de Mattos and de Jager, and also by German authorities, such as Bagynski, concerning the value of buttermilk. Those who have experience of buttermilk agree that as an artificial food for babies, this preparation is more reliable than any other, not only for healthy babies, but also for sickly infants. The increase in weight is more marked, and local intestinal disorders are more favourably influenced.

On earlier occasions I have expressed my opinion with some reserve concerning the use of buttermilk in the case of very young babies (sucklings), but later observations have convinced me that in the case of sucklings also buttermilk yields better results than any other food tried by me. I consider that of the preparations of buttermilk "Lacticia" is of special value, as it will keep indefinitely, and is well adapted for export. The results which I have obtained from its use are identical with those which I obtained with fresh buttermilk of superior quality.

Yours, etc.,

(Dr.) I. Graanboom.

Amsterdam, Weteringschans 98, November, 1908.

